# Activation of Extracellular Signal-Regulated Kinase 2 and cAMP Response Element-Binding Protein in Cultured Neurons by the Macrocyclic Ellagitannin Oenothein B

**DOI:** 10.3390/neurosci3030028

**Published:** 2022-07-07

**Authors:** Satoshi Okuyama, Morio Yoshimura, Yoshiaki Amakura, Mitsunari Nakajima, Yoshiko Furukawa

**Affiliations:** 1 Department of Pharmaceutical Pharmacology, College of Pharmaceutical Sciences, Matsuyama University, 4-2 Bunkyo-cho, Matsuyama 790-8578, Ehime, Japan; sokuyama@g.matsuyama-u.ac.jp (S.O.); mnakajim@g.matsuyama-u.ac.jp (M.N.); 2 Department of Pharmacognosy, College of Pharmaceutical Sciences, Matsuyama University, 4-2 Bunkyo-cho, Matsuyama 790-8578, Ehime, Japan; myoshimu@g.matsuyama-u.ac.jp (M.Y.); amakura@g.matsuyama-u.ac.jp (Y.A.)

**Keywords:** oenothein B, ellagitannin, neurotrophic effect, neuroprotective effect, ERK, CREB, neuron, brain

## Abstract

(1) Background: The findings of our recent in vivo study indicated that the oral administration of oenothein B, a unique macrocyclic ellagitannin, activated extracellular signal-regulated kinase (ERK) 2 and cAMP response element-binding protein (CREB) in the mouse brain. A large hydrophilic oenothein B is unable to reach the brain, suggesting that any metabolite(s) of oenothein B might function in the brain. (2) Results: The addition of oenothein B to the culture medium of rat cortical neurons induced the prompt and significant activation of ERK2 and CREB. (3) Conclusions: The activation of ERK2 and CREB is crucial for synaptic transmission and learning/memory formation in the brain. The present results suggest oenothein B exerts neurotrophic/neuroprotective effects in the brain through the modulation of neuronal signaling pathways, if it reaches the brain.

## 1. Introduction

The benefits of phytochemicals in the treatment/prevention of various diseases and promotion of human health have been examined for many years. Among phytochemicals, polyphenols have attracted attention due to their potent antioxidative activities [1]. They were previously shown to be of therapeutic value in diseases closely associated with active oxygen damage, such as cancer and cardiovascular diseases [2,3]. Recent research efforts revealed that several polyphenols (flavonoids and so on) also exert multiple effects in the nervous system, including the promotion of neurite outgrowth, the enhancement of survival, the activation of tropomyosin receptor kinases (Trk), and the induction of neurotrophic factor expression in neuronal cells [4,5,6], which are mediated by neurotrophic signaling pathways [7].

**Figure 1:**
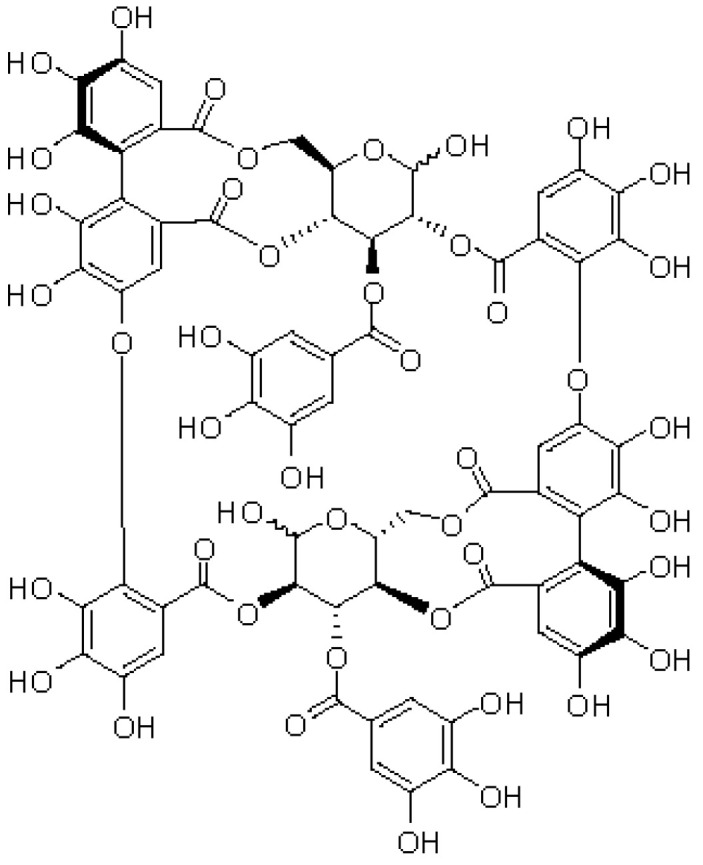
Structure of oenothein B. Oenothein B comprises two tellimagrandin I monomers linked between hexahydroxydiphenoyl groups and galloyl groups on the glucopyranose ring. Its molecular weight is 1568.

Oenothein B (Figure 1), which is grouped into the ellagitannins, is also a polyphenol with higher molecular weight [8]. Many studies have revealed that oenothein B exhibits various biological activities including antioxidant, anti-inflammatory, antitumor, antibacterial and so on, and that it confers health benefits to humans [9,10]. In addition to these previously reported effects on peripheral cells/tissues, we recently demonstrated that oenothein B acts on the brain [11,12]. We showed in in vivo studies that (1) the oral administration of oenothein B to systemic inflammatory model mice reduced the inflammatory response in the brain and suppressed abnormal behavior [11], and (2) the oral administration of oenothein B to healthy mice activated (phosphorylated) extracellular signal-regulated kinase (ERK) 2 and cAMP response element-binding protein (CREB) in the hippocampus, a part of the brain that is involved in learning/memory formation [12]. The latter findings suggested that oenothein B exerts neurotrophic/neuroprotective effects in the brain because the phosphorylated ERK2 (pERK2)/ERK2 ratio and phosphorylated CREB (pCREB)/CREB ratio in the hippocampus were both shown to be significantly elevated by synaptic transmission and learning/memory formation [13,14] and CREB is a transcription factor for some neurotrophic factors, such as brain-derived neurotrophic factor (BDNF) [15]. We speculated that in the latter case [12], any metabolite(s) of oenothein B (for example, urolithins) function in the brain because oenothein B, a large hydrophilic molecule, may be unable to reach the brain as it is [16]. Therefore, we are now investigating the urinary and plasma metabolite(s) of oenothein B. 

There are reports that (1) ellagitannins are fairly stable in the stomach and are likely to act directly in situ in this area [17], and (2) biological actions of oenothein B require its full molecular structure, as substructures of oenothein B such as pyrocatechol, gallic acid, pyrogallol, 3,4-dihydroxybenzoic acid were all inactive [18]. It can be expected that oenothein B itself or some of its slightly modified forms reach the brain when the blood-brain barrier becomes injured and that oenothein B is beneficial to prevent the deterioration of learning and memory ability in the case of cerebral ischemia or trauma. Although we have not yet actually confirmed the existence of oenothein B in the brain, we herein examined its effects on ERK2 and CREB in cultured neurons because we were convinced that water-soluble oenothein B can directly act on neurons, as in the case of peripheral immune cells [19,20].

## 2. Materials and Methods

### 2.1. Preparation of Oenothein B and Reagents

Oenothein B was isolated from the leaves of *Eucalyptus globulus* as previously described [21]. Reversed phase HPLC of oenothein B showed multiple peaks (*t*_R_ 21–26 min) due to major and minor anomers at two glucose cores in a molecule. The purity of the sample used in the present study was estimated to be >95% based on the relative ratio (%) of the total area of major and minor peaks to that of the same concentration of exhaustively purified oenothein B (as the standard) in the HPLC chromatogram.

### 2.2. Cell Cultures

Primary cultures of cortical neurons were prepared from Wistar rats (Charles River Laboratories Japan, Inc., Yokohama, Japan) on embryonic day 18, as previously described [22]. Cells obtained by the enzymatic digestion of tissues by trypsin/DNase were suspended in Dulbecco’s modified Eagle’s medium (Sigma-Aldrich Company Ltd., St. Louis, MO, USA) containing 5% fetal bovine serum (ICN Biochemicals, Aurora, OH, USA) and seeded on culture vessels precoated with poly DL-ornithine (Sigma). To assess cell viability, cells were seeded on 96-well plates at a density of 3.7 × 10^4^ cells/well. In an immunoblot analysis, cells were seeded on 6-well plates at a density of 10^6^ cells/well. After a 24 h culture period, the medium was changed to Neurobasal medium (Invitrogen Corp., Carlsbad, CA, USA) containing B27 supplement (Invitrogen) and 2 mM glutamine, and cells were then cultured for 3 days. Cells were incubated for the desired times with the test compounds (oenothein B or BDNF), which were dissolved in dimethyl sulfoxide (DMSO). The final concentration of DMSO in all culture media was <0.1%.

### 2.3. Assessment of Cell Viability

Cellular viability was assessed by the 3-[4,5-dimethylthiazol-2-yl]-2,5-diphenyl-tetrazolium bromide (MTT) assay as previously described [23]. MTT was purchased from Sigma-Aldrich.

### 2.4. Western Blot Analysis

Protein extracts of cells were prepared as previously described [22]. Proteins (10 μg) in each extract were separated on an SDS-polyacrylamide gel and electroblotted onto an Immuno-Blot^TM^ PVDF Membrane (BIO-RAD Laboratories, Hercules, CA, USA). Antibodies and their sources were as follows: a rabbit antibody against MAP kinase 1/2 (Erk1/2-CT), which recognizes ERK1 and ERK2, from Upstate Biotechnology, Inc. (Lake Placid, NY, USA); a rabbit antibody against phospho-p44/42 MAP Kinase, which recognizes phosphorylated ERK1 (Thr-202 and Tyr-204) and phosphorylated ERK2 (Thr-185 and Tyr-187); a rabbit antibody against CREB; a rabbit antibody against phosphorylated CREB (Ser-133); and horseradish peroxidase-linked anti-rabbit IgG from Cell Signaling Technology (Woburn, MA, USA). Fluorescence-based Western blotting detection reagents were purchased from Amersham Biosciences Corp. (Piscataway, NJ, USA).

### 2.5. Statistical Analysis

All results were expressed as means ± SEM. Experiments involving 3 or more groups were subjected to a one-way ANOVA followed by Dunnett’s multiple comparison test (Prism 6; GraphPad Software, La Jolla, CA, USA). *p* < 0.05 was considered to be significant.

## 3. Results

ERK2, a serine/threonine protein kinase belonging to the mitogen-activated protein kinase (MAPK) family, is one of the typical ERKs along with ERK1. Since ERK2 (42 kDa) and ERK1 (44 kDa) exhibit 85% sequence homology, the majority of antibodies against ERK2 recognize ERK1. Furthermore, ERK1 and ERK2 are coordinately activated (i.e., phosphorylated) when stimulated [24]. Therefore, they are generally recognized as ERK1/2. Although ERK1/2 signaling has been implicated in many diverse cellular events in various cells, ERK2, not ERK1, signaling is involved in neurogenesis and cognitive function in the central nervous system (CNS) [13]. Therefore, we examined the ratio of pERK2/ERK2 but not pERK1/ERK1. BDNF, one of the representative neurotrophic factors in the CNS, was used as the positive control for activators of ERK2 and CREB. 

**Figure 2:**
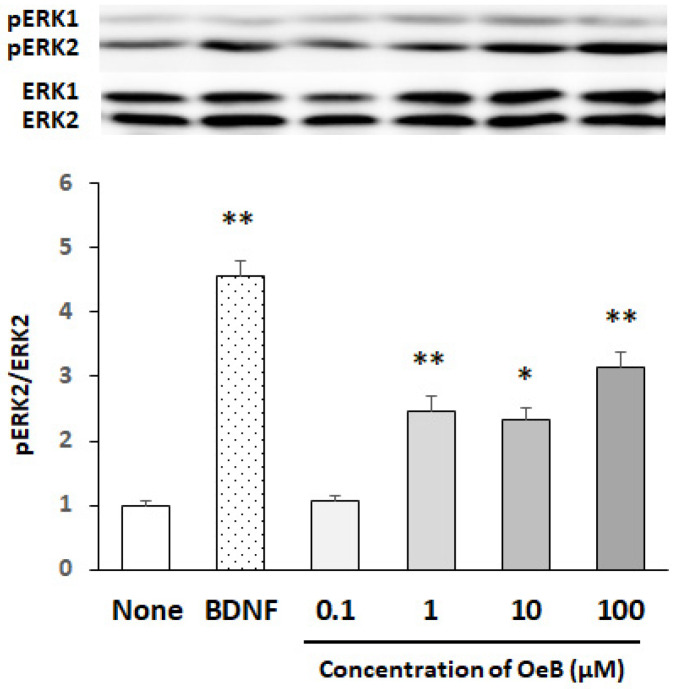
Dose-dependent phosphorylation of ERK2 after the treatment of cultured rat cortical neurons with oenothein B. Cells were treated with various concentrations (0.1, 1, 10, and 100 μM) of oenothein B (OeB) for 30 min or BDNF (50 ng/mL) for 10 min. The density ratio of phosphorylated components to total components (pERK2/ERK2) of untreated cultures (None) was expressed as 1. Results are given as means ± SEM (*n* = 4). Significance differences between compound-treated and untreated cells: * *p* < 0.05, ** *p* < 0.01.

We initially examined the concentration dependency of oenothein B for the activation (i.e., phosphorylation) of ERK2 in cultured neurons. Since the activation of ERK1/2 was previously shown to be promptly induced by various stimuli [25], the reaction time was set to 30 min. To eliminate the possibility that cells were damaged during the treatment with oenothein B, we investigated the effects of a 30 min exposure to oenothein B on cell viability using the MTT assay. The results obtained showed no significant differences in cell viability between non-treated cells and cells incubated with oenothein B even at a concentration of 100 μM (data not shown). Therefore, we set the dose range of oenothein B to 0.1–100 μM. Figure 2 shows representative bands of a Western blot for pERK1/2 and ERK1/2 and the ratios of pERK2 to ERK2 (untreated cultures were expressed as one arbitrary unit) in cultured neurons, which indicated that a significant increase in the pERK2/ERK2 ratio was detectable over the concentration range of 1–100 μM. Figure 2 also shows that oenothein B markedly phosphorylated ERK2, similar to BDNF, which induced the rapid (within 10 min) and potent phosphorylation of ERK2.

**Figure 3:**
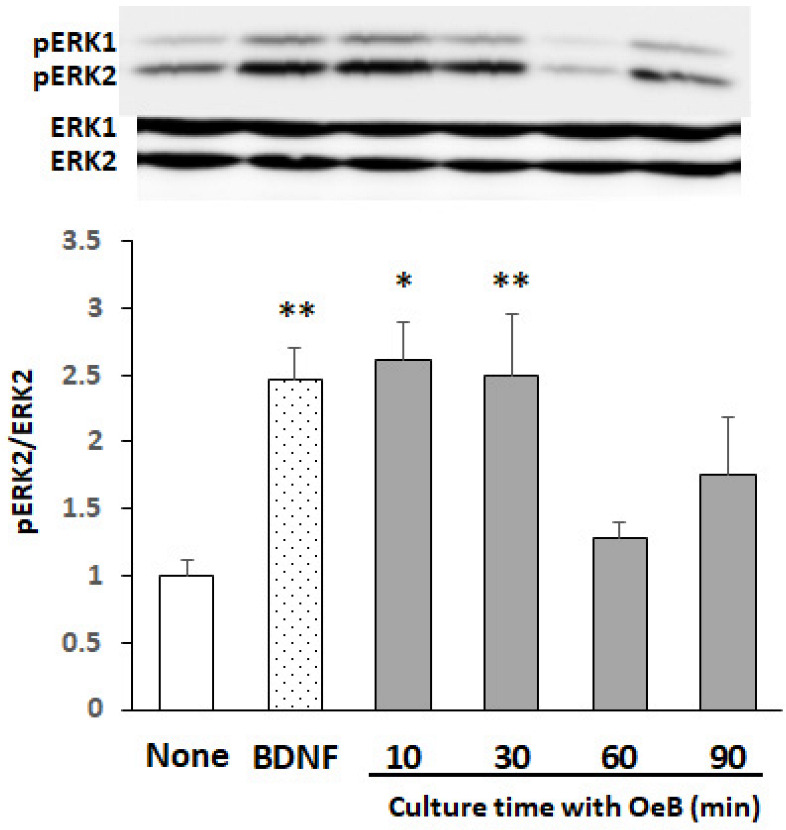
Time-dependent phosphorylation of ERK2 after the treatment of cultured rat cortical neurons with oenothein B. Cells were treated with 50 μM oenothein B (OeB) for various times (10, 30, 60 and 90 min) or with 50 ng/mL BDNF for 10 min. The density ratio of phosphorylated components to total components (pERK2/ERK2) in untreated cultures (None) was expressed as 1. Results are given as means ± SEM (*n* = 4). Significance differences between compound-treated and untreated cells: * *p* < 0.05, ** *p* < 0.01.

To examine the time-dependent responses of cultured neurons to oenothein B based on the phosphorylation of ERK2, we incubated neurons with 50 μM oenothein B for 10–90 min. Figure 3 shows the representative bands of a Western blot for pERK1/2 and ERK1/2, and pERK2/ERK2 ratios in cultured neurons. Significant increases were observed at 10 and 30 min, followed by a decrease to the control level 60 min after the start of the oenothein B treatment. These results demonstrated that oenothein B rapidly phosphorylated ERK2, similar to BDNF.

**Figure 4:**
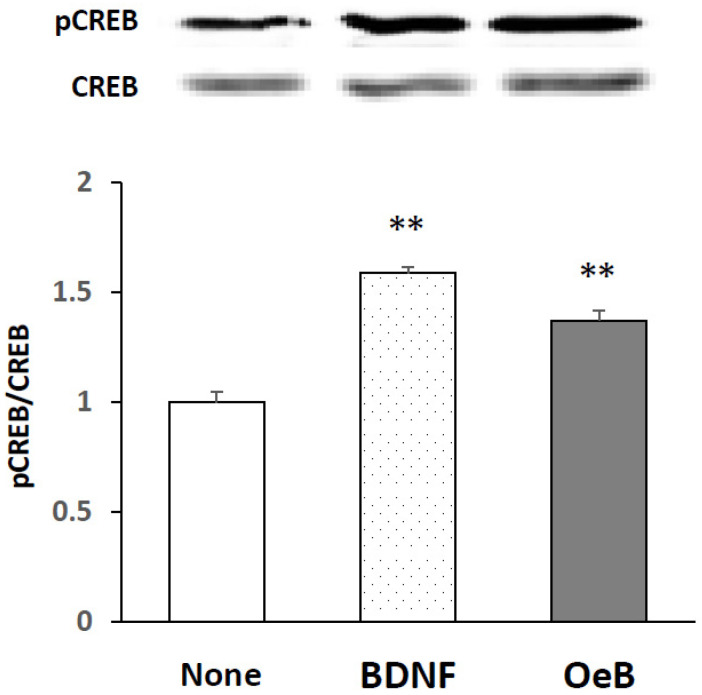
Effects of oenothein B on CREB activation in rat cortical neurons. Cells were incubated with or without oenothein B (OeB) at a concentration of 100 μM for 30 min or with 50 ng/mL BDNF for 10 min. The density ratio of phosphorylated components to total components (pCREB/CREB) in untreated cells (None) was expressed as 1. Values are presented as means ± SEM (*n* = 3). Significant difference between compound-treated and untreated cells: ** *p* < 0.01.

To investigate whether oenothein B induced the activation of CREB, we cultured cells with 100 μM oenothein B for 30 min. Figure 4 shows the representative bands of a Western blot for pCREB and CREB and the pCREB/CREB ratio in cultured neurons, indicating that the treatment with oenothein B significantly phosphorylated CREB, similar to BDNF (at a concentration of 50 ng/mL for 10 min).

## 4. Discussion

The present results showed that oenothein B acted as an activator of ERK2 and CREB in cultured neurons, suggesting the possibility that oenothein B might show neurotrophic/neuroprotective effects, if it reaches the brain in its original form. The present result in Figure 2 demonstrates that oenothein B induces a significant increase in the pERK2/ERK2 ratio at the concentration of 1–100 μM. This result matches substantially to a previous report showing that ellagitannins have some relevant anti-atherogenic, anti-thrombotic, anti-inflammatory and anti-angiogenic effects at concentrations of 10–100 μM in in vitro studies [26].

Recent research efforts have contributed to the accumulation of evidence showing that several polyphenols (flavonoids such as nobiletin, and nonflavonoids such as auraptene and curcuminoids) exert neurotrophic/neuroprotective effects via various signaling pathways, including the ERK1/2, phosphoinositide-3 kinase/Akt and phospholipase Cγ/protein kinase C pathways [7]. Our research group demonstrated that 3,5,6,7,8,3′,4′-heptamethoxyflavone, a polymethoxyflavone and one of the representative citrus polyphenols, exerted neurotrophic/neuroprotective effects in vitro [22,23,27] and in vivo [22,28,29,30,31,32,33] via the cAMP/ERK/CREB signaling pathway [23]. Although we have not yet investigated whether oenothein B activates signaling pathways other than the ERK2 signaling pathway, we speculate that oenothein B exerts its effects in the CNS via at least the ERK2 signaling pathway.

The present result in Figure 3 demonstrates that oenothein B rapidly activates ERK2. This result suggests that oenothein B acts on any receptor, followed by the activation of the ERK2 signaling pathway. It cannot be assumed that the activation of the ERK2 signaling pathway by oenothein B is mediated by its antioxidant ability, because it might require a long time to have an effect in that case. It is reasonable to consider that a large water-soluble molecule, oenothein B, which is unable to be taken into cells, might act on any receptor on the cell surface, and consequently activate the ERK2 signaling pathway. We previously reported that 4-methylcatechol (4-methylbenzene-1,2-diol), a synthetic compound with two phenolic hydroxy residues, stimulated the phosphorylation of ERK1/2 along with the BDNF receptor Trk B [34]. Furthermore, another group demonstrated that nobiletin, which exerts memory-improving effects in various animal models of dementia, enhanced protein kinase A (PKA)/ERK/CREB signaling in PC12D cells and cultured rat hippocampal neurons and induced long-term potentiation by activating the PKA-dependent phosphorylation of the alpha-amino-3-hydroxy-5-methyl-4-isoxazolepropionic acid receptor subunit GluR1 in hippocampus slices [35]. We are now investigating which receptors trigger the activation of ERK2 and the continual activation of CREB by oenothein B.

The present result in Figure 4 demonstrates that oenothein B induces the prompt and significant activation of CREB. We speculate that oenothein B induces phosphorylation of CREB via phosphorylation of ERK2. In order to clarify this possibility, we will investigate whether a specific inhibitor of MAPK/ERK kinase 1 (such as U0126) has the ability to inhibit CREB-phosphorylation by oenothein B.

In conclusion, the addition of oenothein B to the culture medium of rat cortical neurons induced the prompt and significant activation of ERK2 and CREB. Active attempts are being made to apply polyphenols to the treatment of neurodegenerative disorders, such as Alzheimer’s disease, Parkinson’s disease, and Huntington’s disease [36,37,38,39]. The present results and our previous findings [12] collectively suggest that oenothein B and possibly plant materials containing oenothein B are useful for the treatment of neurological disorders.

## Data Availability

The data presented in this study are available within the article.
